# Enhancing crop innate immunity: new promising trends

**DOI:** 10.3389/fpls.2014.00624

**Published:** 2014-11-06

**Authors:** Pin-Yao Huang, Laurent Zimmerli

**Affiliations:** ^1^Department of Life Science, National Taiwan UniversityTaipei, Taiwan; ^2^Institute of Plant Biology, National Taiwan UniversityTaipei, Taiwan

**Keywords:** innate immunity, pattern-triggered immunity, pattern-recognition receptor, leucine-rich repeat receptor kinase, lectin receptor kinase, pathogen, microbe

## Abstract

Plants are constantly exposed to potentially pathogenic microbes present in their surrounding environment. Due to the activation of the pattern-triggered immunity (PTI) response that largely relies on accurate detection of pathogen- or microbe-associated molecular patterns by pattern-recognition receptors (PRRs), plants are resistant to the majority of potential pathogens. However, adapted pathogens may avoid recognition or repress plant PTI and resulting diseases significantly affect crop yield worldwide. PTI provides protection against a wide range of pathogens. Reinforcement of PTI through genetic engineering may thus generate crops with broad-spectrum field resistance. In this review, new approaches based on fundamental discoveries in PTI to improve crop immunity are discussed. Notably, we highlight recent studies describing the interfamily transfer of PRRs or key regulators of PTI signaling.

## INTRODUCTION

Monocultures of highly fertilized crops that are typical of intensive agriculture practices are very sensitive to disease by adapted pathogens (Bruce, 2012). The development of resistant crops is thus critical for better yields. Although prone to disease, plants have evolved diverse defense mechanisms to cope with potential pathogens. To sense invaders, plants are equipped with surveillance machineries such as plasma membrane surface-localized proteins called pattern recognition receptors (PRRs), which detect foreign (non-self) pathogen- or microbe-associated molecular patterns (PAMPs or MAMPs; Boller and Felix, 2009; Bohm et al., 2014; Zipfel, 2014), as well as self-derived damage-associated molecular patterns (DAMPs; Boller and Felix, 2009; Newman et al., 2013; Zipfel, 2014). MAMPs are evolutionarily conserved across a certain class of microbes and are essential for the microbial lifestyle. Some examples of bacterial MAMPs and their corresponding PRRs include flagellin/FLAGELLIN SENSING2 (FLS2; Gómez-Gómez and Boller, 2000), EF-Tu/EF-Tu RECEPTOR (EFR; Zipfel et al., 2006), *Xanthomonas* eMAX/RECEPTOR OF eMAX (ReMAX; Jehle et al., 2013) and peptidoglycan (PGN)/LYSIN-MOTIF1 (LYM1) and LYM3 (Willmann et al., 2011). Fungal MAMPs/PRRs pairs are exemplified by chitin/CHITIN ELICITOR RECEPTOR KINASE1 (CERK1; Miya et al., 2007; Wan et al., 2008), xylanase/ETHYLENE INDUCING XYLANASE2 (Eix2; Ron and Avni, 2004), and avirulence gene Ave1/VERTICILIUM1 (Ve1; de Jonge et al., 2012). DAMPs are endogenous molecules released upon cell damage or pathogen recognition. The known DAMPs/PRRs pairs include Pep peptides/PEP1 RECEPTOR 1 (PEPR1) and PEPR2 (Huffaker et al., 2006; Yamaguchi et al., 2006; Krol et al., 2010; Yamaguchi et al., 2010), cell wall fragment oligogalacturonides (OGs)/WALL-ASSOCIATED KINASE 1 (Brutus et al., 2010), and extracellular ATP (eATP)/DOES NOT RESPOND TO NUCLEOTIDES 1 (DORN1; Choi et al., 2014).

The recognition of MAMPs or DAMPs by PRRs activates the pattern-triggered immunity (PTI) response (Tsuda and Katagiri, 2010). Increased cellular Ca^2+^ concentration, production of reactive oxygen species (ROS), and activation of mitogen-activated protein kinase (MAPK) cascades are considered as early PTI responses, whereas callose deposition and marker gene up-regulation are observed later during PTI (Boller and Felix, 2009; Zipfel and Robatzek, 2010; Tena et al., 2011). These first layers of defense are believed to be sufficient to prevent the invasion of a wide range of pathogens. Functional PRRs are crucial for the success of PTI, as a defective recognition system makes plants more vulnerable to their surrounding environment. Notably, loss-of-function mutations in *FLS2* impair *Arabidopsis thaliana* resistance against *Pseudomonas syringae* pv. *tomato* (*Pst*) DC3000 bacteria (Zipfel et al., 2004) and *Arabidopsis efr* mutants show increased susceptibility to *Agrobacterium tumefaciens* (Zipfel et al., 2006). Similarly, *Arabidopsis cerk1* mutants display enhanced sensitivity to fungal pathogens (Miya et al., 2007; Wan et al., 2008), and *Arabidopsis pepr1 pepr2* plants are more susceptible than wild-type plants to *Pst* DC3000, *Botrytis cinerea*, and *Colletotrichum higginsianum* (Ma et al., 2012; Liu et al., 2013; Ross et al., 2014). In addition to PRRs, other regulators are required for full activation of PTI (Macho and Zipfel, 2014). For example, BRI1-ASSOCIATED RECEPTOR-LIKE KINASE/SOMATIC EMBRYOGENESIS RECEPTOR-LIKE KINASE3 (BAK1/SERK3) acts as a co-receptor for the conserved 22-amino acid epitope flg22 of the bacterial flagellin, and forms a complex with FLS2 upon flg22 perception (Chinchilla et al., 2007; Sun et al., 2013). BOTRYTIS-INDUCED KINASE1 (BIK1) is also critical for flg22-mediated signal transduction from the FLS2/BAK1 receptor complex (Lu et al., 2010; Zhang et al., 2010). Accordingly, loss-of-function mutants of *BAK1* or *BIK1* display impaired flg22-induced responses (Chinchilla et al., 2007; Heese et al., 2007; Lu et al., 2010; Zhang et al., 2010). Recent studies showed that L-type lectin receptor kinases (LecRKs) modulate the PTI response (Singh and Zimmerli, 2013). LecRK-I.9, also known as DORN1, is necessary for eATP recognition, and is required for MAMP-induced callose deposition (Bouwmeester et al., 2011a; Choi et al., 2014). In addition, LecRK-V.5 negatively regulates MAMP-induced ROS burst in guard cells (Desclos-Theveniau et al., 2012), and LecRK-VI.2 associates with FLS2 and positively modulates PTI upstream of MAPK signaling (Singh et al., 2012a; Huang et al., 2014a).

Though PTI is sufficient to limit the proliferation of a wide variety of microbes, successful pathogens often break plant resistance via delivering virulence molecules called effectors into the apoplast or host cells to suppress PTI (Dou and Zhou, 2012). As a counter measure, plants deploy resistance (R) proteins that generally perceive directly or indirectly perturbations of effectors to mount another layer of defense called effector-triggered immunity (ETI; Jones and Dangl, 2006; Dodds and Rathjen, 2010). ETI is characterized by the induction of a strong and transient immune response often correlated with localized cell death to restrict pathogen spread (Jones and Dangl, 2006). However, rapidly evolving pathogens are able to overcome ETI via frequent mutations in effectors, escaping host R protein detection (Gassmann et al., 2000; Jones and Dangl, 2006; Huang et al., 2014b).

It is a slow process to generate disease resistant crop varieties via traditional breeding, which involves crossing between different varieties and multiple backcrossing to select progenies with the most positive and least negative traits. With the advances in genetic engineering, novel basic knowledge on plant immunity can be applied successfully toward the rapid development of disease resistant crops. To combat crop diseases, relevant defense-related genes can be transferred from one plant species to another. Interspecies or interfamily gene transfer has been shown to be feasible with heterologous genes remaining functional after transfer (Wulff et al., 2011; Dangl et al., 2013). Detailed molecular mechanisms are however not yet well understood. The compatibility of gene transfer across plant species/families suggests that components of defense signaling pathways are conserved between species. In agreement with this, MAPK cascades are crucial for various defense responses in *Arabidopsis*, tomato, *Nicotiana benthamiana*, and rice (Rodriguez et al., 2010; Meng and Zhang, 2013). Similarly, the plant ROS burst represents a general hallmark of pathogen recognition and defense activation (Torres, 2010).

With the increasing number of plant defense regulators identified, many examples have been established to test the efficacy of heterologous gene transfer on enhancing disease resistance. In this review, we discuss recent findings on improving plant immunity via transfer of defense-related genes, with a special focus on approaches exploiting PTI to confer broad-spectrum resistance.

## RATIONALES FOR PTI-BASED BIOENGINEERING

Strategies to improve crop immunity via transfer of *R* genes were extensively used in the past decades (Wulff et al., 2011; Dangl et al., 2013). However, the durability of *R* gene-mediated resistance can be greatly hampered by the rapid evolution of pathogen effectors, which are only partially critical for pathogen fitness and virulence (Gassmann et al., 2000; Zhou et al., 2007; Ayliffe et al., 2008; Huang et al., 2014b). Moreover, effectors are species, race, or strain specific, making it difficult to combat diverse pathogens with a single *R* gene transfer (Chisholm et al., 2006; Jones and Dangl, 2006; Bent and Mackey, 2007; Thomma et al., 2011). By contrast, MAMPs, which are conserved within a class of microbes, are less likely to adopt mutations since they are crucial for the fitness and survival of microbes. For example, the MAMP flagellin from bacterial flagella is critical for bacterial motility, and peptidoglycans are inherent of the cell wall of Gram-positive bacteria (Felix et al., 1999; Nürnberger et al., 2004; Zipfel and Felix, 2005; Gust et al., 2007; Erbs et al., 2008). Similarly, DAMPs, which serve as common danger signals released from stressed-cells, induce plant immune responses against a large variety of pathogens (Huffaker et al., 2006; Yamaguchi et al., 2010; Liu et al., 2013). Accordingly, approaches exploiting PTI may stand a better chance in engineering crops with durable resistance against diverse pathogens.

## GAIN OF NEW MAMP PERCEPTION CAPABILITIES

Recognition of MAMPs by PRRs is the first step in PTI activation and consequently, plants defective in MAMP recognition are more susceptible to pathogens. Conversely, the introduction of a new PRR to a given plant species may boost PTI responses via additional PTI activation signaling from the new MAMP/PRR recognition system. This experimental hypothesis was demonstrated to be feasible through the interfamily transfer of EFR, a bacterial EF-Tu receptor (Lacombe et al., 2010). EF-Tu is one of the most abundant, widely conserved, and slow-evolving protein in bacteria (Lathe and Bork, 2001; Kunze et al., 2004; Lacombe et al., 2010). Recognition of EF-Tu (or its eliciting epitope elf18) is *Brassicaceae* specific (Kunze et al., 2004; Zipfel et al., 2006) and *Solanaceous* plants such as *N. benthamiana* and tomato do not possess EFR (Kunze et al., 2004). Remarkably, *N. benthamiana* and tomato plants with stable expression of *EFR* gain responsiveness to elf18 as illustrated by the accumulation of ROS and PTI-responsive mRNAs (Lacombe et al., 2010). Moreover, heterologous expression of *EFR* in *N. benthamiana* and tomato greatly increases resistance toward a wide range of pathogens carrying the eliciting EF-Tu (Lacombe et al., 2010). Transgenic expression of *EFR* in *N. benthamiana* and tomato does not result in constitutive defense responses or defects in growth and development when assessed in laboratory conditions (Lacombe et al., 2010), fulfilling basic agronomical requirements. Theoretically, host co-evolved pathogens are unlikely to possess effectors that target the new PRR signaling originally absent from the host (Lacombe et al., 2010), making PRR genetic engineering a promising tool in agricultural biotechnology. Similarly, *N. benthamiana* plants lacking ReMAX, the PRR for perception of the MAMP eMAX respond to eMAX treatment when a chimeric receptor engineered from ReMAX and the tomato Eix2 is transiently expressed (Jehle et al., 2013). It would be interesting to test whether stable transformation of *N. benthamiana* with ReMAX could confer resistance to a wide range of *Xanthomonas* bacteria. *Xanthomonas* bacteria are indeed known to cause serious diseases in major crops, and effective strategies are required to control such diseases (Ryan et al., 2011). Bacterial blight of rice, caused by *Xanthomonas oryzae* pv. *oryzae* (*Xoo*) is one of the most devastating disease in rice (Nino-Liu et al., 2006). Transfer of the potential PRR XA21 (Song et al., 1995) from wild rice *Oryza longistaminata* to the susceptible rice cultivar (*Oryza sativa* subsp. japonica var. Taipei 309) confers resistance to multiple isolates of *Xoo* (Wang et al., 1996), suggesting that XA21 can be used as a tool to control rice blight. Similarly, *Xanthomonas campestris* pv. *musacearum* (*Xcm*) causes banana *Xanthomonas* wilt (BXW) and has a huge impact on banana yield (Tripathi et al., 2009). Comparative genomic analysis between *Xoo* and *Xcm* revealed a conserved set of bacterial genes required for the activation of XA21-mediated immunity, suggesting that XA21 can be used for engineering resistance against *Xcm* (Tripathi et al., 2014). This hypothesis was confirmed by the evaluation of transgenic banana plants expressing *Xa21* for BXW resistance (Tripathi et al., 2014). After inoculation of *Xcm*, non-transgenic banana plants display typical BXW symptoms such as yellow ooze in pseudostem, spreading of *Xcm*, and complete wilting, whereas transgenic banana plants expressing *Xa21* show only few or no symptoms, indicating enhanced resistance (Tripathi et al., 2014). Like in tomato plants expressing *EFR*, growth is not altered in banana plants expressing *Xa21* (Tripathi et al., 2014). Interestingly, transfer of XA21 to dicot plants such as sweet orange (*Citrus sinensis*) or tomato also confers resistance against *Xanthomonas axonopodis* pv. *citri* and *Ralstonia solanacearum*, respectively (Mendes et al., 2010; Afroz et al., 2011). XA21 thus stands as a promising candidate for engineering resistance against diverse pathogens in different plant species.

## BOOSTING THE PTI RESPONSE

LecRKs belong to a class of receptor kinases characterized by an extracellular lectin domain, and are involved in plant development and stress responses (Bouwmeester and Govers, 2009; Vaid et al., 2012; Singh and Zimmerli, 2013). Although the lectin motif is suggested to bind to oligosaccharides or small hydrophobic ligands (Barre et al., 2002; Andre et al., 2005; Bouwmeester and Govers, 2009), a recent study showed that *Arabidopsis* LecRK-I.9/DONR1 acts as a PRR for the DAMP eATP (Cao et al., 2014; Choi et al., 2014). In mammalian cells, abnormal or uncontrolled increase of eATP represents a danger signal from damaged or stressed cells, and is involved in activating the innate immune system (Hanley et al., 2004). Similarly, plant eATP is increased upon various stresses and is proposed to play a central role in regulating plant immunity (Tanaka et al., 2010; Cao et al., 2014; Choi et al., 2014). Importantly, *Arabidopsis lecrk-I.9/dorn1* displays impaired ATP-triggered PTI responses, such as Ca^2+^ influx, activation of MAPK, and up-regulation of stress-induced genes (Choi et al., 2014). LecRK-I.9/DONR1 was initially identified as a target of the *Phytophthora infestans* RXLR-dEER effector IPI-O (Gouget et al., 2006). LecRK-I.9/DONR1 also contributes to *Arabidopsis* resistance against *Phytophthora brassicae*, and is important for maintaining cell wall (CW)-plasma membrane (PM) continuum (Bouwmeester et al., 2011a). Ectopic expression of *LecRK-I.9/DONR1* in *Solanaceous* potato and *N. benthamiana* plants results in enhanced resistance against *Phytophthora infestans* (Bouwmeester et al., 2014). The CW-PM continuum is hypothesized to be critical for guarding pathogen invasion, and virulent pathogens destabilize through effector action the CW-PM continuum to facilitate colonization (Bouwmeester et al., 2011a,b). The enhanced resistance observed in transgenic potato and *N. benthamiana* may thus be the result of a strengthening of CW-PM adhesions by ectopic expression of *LecRK-I.9/DONR1* (Bouwmeester et al., 2011a,b, 2014). Alternatively, heterologous expression of *LecRK-I.9/DONR1* may trigger an enhanced PTI response via perception of eATP released from pathogen-stressed cells (Choi et al., 2014). When grown in greenhouse condition, stable transgenic potato lines expressing *LecRK-I.9/DONR1* display aberrant plant development including wrinkled leaves, decreased leaflet separation, and malformed tuber (Bouwmeester et al., 2014). The strengthening of CW-PM adhesion by heterologous expression of *LecRK-I.9/DONR1* may disrupt normal plant development (Bouwmeester et al., 2014).

**FIGURE 1:**
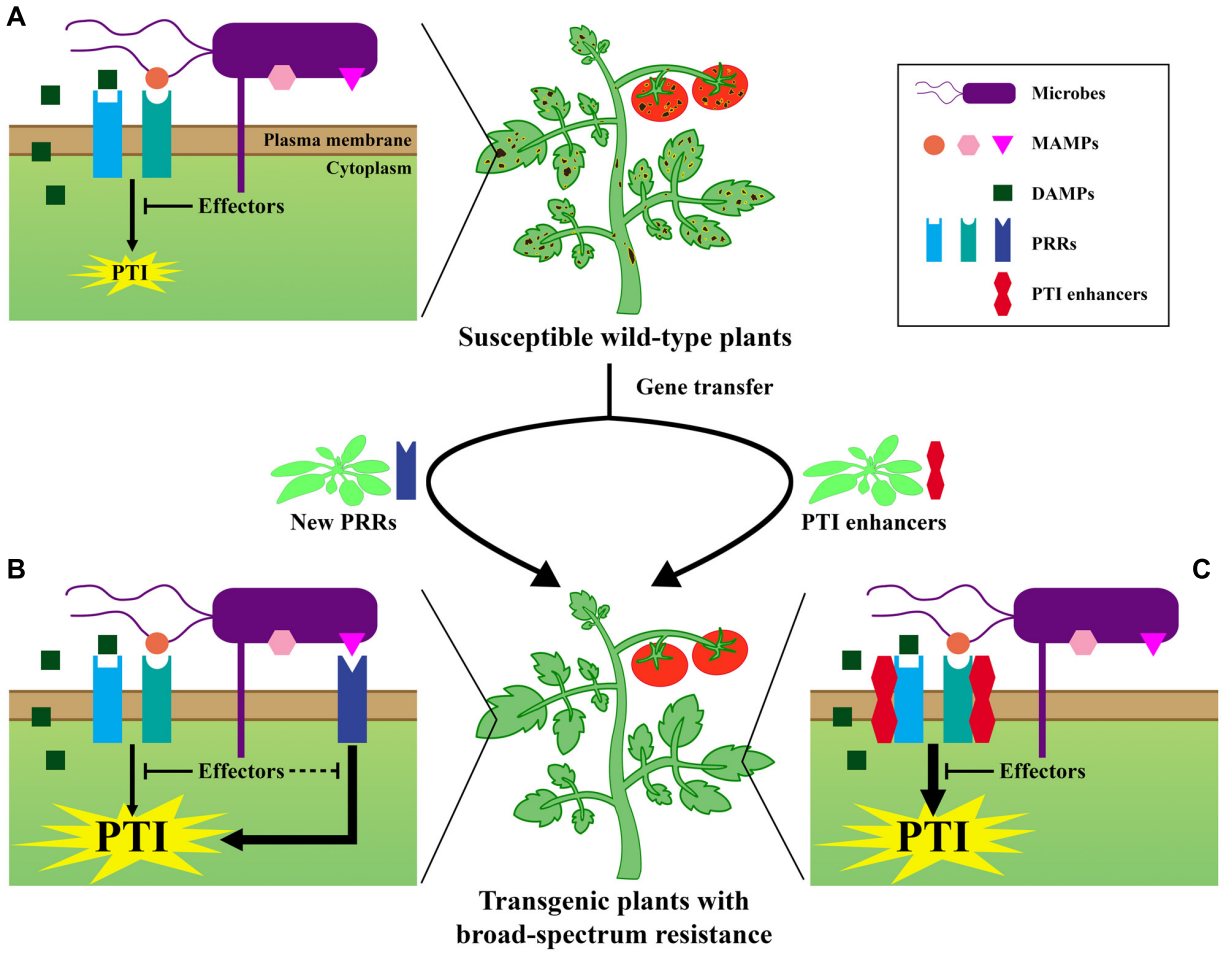
**Pattern-triggered immunity (PTI) mediated strategies to deploy broad-spectrum pathogen resistance in crops: a conceptual representation. (A)** Perception of MAMPs and/or DAMPs by PRRs activates the PTI response. Adapted, virulent pathogens deliver effectors to suppress PTI, rendering wild-type plants susceptible to infection. **(B)** Interfamily or interspecies transfer of foreign PRRs improves MAMPs recognition and further enhances PTI. Host co-evolved pathogens are unlikely to possess effectors targeting PRRs from other plant species. **(C)** Heterologous expression of PTI enhancers/positive regulators boosts PTI signaling. Effectors may thus not be sufficient anymore to efficiently repress PTI.

*Arabidopsis* LecRK-VI.2 was first identified as being involved in ABA inhibition of seed germination (Xin et al., 2009), and was later shown to be a component of the FLS2 complex positively regulating PTI (Singh et al., 2012a; Huang et al., 2014a). *Arabidopsis* plants over-expressing *LecRK-VI.2* demonstrate a constitutively activated PTI, and display significant resistance against hemi-biotrophic *Pst* DC3000 and necrotrophic *Pectobacterium carotovorum* ssp. *carotovorum* (*Pcc*) SCC1 bacteria (Singh et al., 2012a). *Arabidopsis* plants over-expressing *LecRK-VI.2* demonstrate a dwarf phenotype (Singh et al., 2012a), as already observed in plants with constituve defense responses (Bowling et al., 1994; Li et al., 2001). LecRK-VI.2-mediated resistance in the *Brassicaceae* plant *Arabidopsis* can be extended to the *Solanaceous* family, as heterologous expression of *Arabidopsis LecRK-VI.2* in *N. benthamiana* enhances wild tobacco resistance against two strains of hemi-biotrophic *Pseudomonas* bacteria and to necrotrophic *Pcc* SCC1 bacteria (Huang et al., 2014a). Remarkably, even 2 weeks after inoculation with *Pseudomonas syringae* pv. *tabaci* 11528, *N. benthamiana* plants expressing *LecRK-VI.2* harbor only weak disease symptoms and normal development of flowers, whereas wild-type (WT) and empty Vector control plants are extremely stunted, and display severe necrotic symptoms with no flowering (Huang et al., 2014a). In line with what is observed in *Arabidopsis* (Singh et al., 2012b), *LecRK-VI.2* protective effect in *N. benthamiana* is bacteria specific (Huang et al., 2014a). However, heterologous expression of *LecRK-VI.2* in *N. benthamiana* does not directly activate, but only potentiates flg22-induced PTI responses. Priming of PTI may explain the observed enhanced resistance in transgenic *N. benthamiana* plants (Conrath et al., 2006; Conrath, 2011; Huang et al., 2014a). These emerging examples of heterologous expression of PRRs or of modulators of PRRs that can confer broad-spectrum resistance through a potentiated PTI response represent an interesting proof of concept approach that suggest feasibility for future applications to engineer resistant crops through primed PTI (**Figure 1**; Lacombe et al., 2010; Huang et al., 2014a). Similarly to transgenic expression of *EFR* (Lacombe et al., 2010), *N. benthamiana* plants expressing *Arabidopsis LecRK-VI.2* demonstrate a WT growth pattern under laboratory conditions (Huang et al., 2014a). The WT-like growth phenotype in *N. benthamiana* as opposed to the stunted phenotype observed in *Arabidopsis* may result from partial conservation of downstream PTI signaling in *N. benthamiana* (Huang et al., 2014a).

In *Arabidopsis*, LecRK-VI.2 is crucial for the up-regulation of PTI marker genes responsive to numerous bacterial MAMPs such as flg22, elf18, PGN, and lipopolysaccharide (Singh et al., 2012a), that are recognized by different PRRs (Gómez-Gómez and Boller, 2000; Zipfel et al., 2006; Willmann et al., 2011). In addition to associate with the PRR FLS2 (Huang et al., 2014a), LecRK-VI.2 may thus prime the PTI response through association and positive action at additional, different PRR complexes. Therefore, heterologous expression of *LecRK-VI.2* is likely to confer broad-spectrum resistance in other plant species via targeting of multiple PRRs. Therefore, *Arabidopsis* LecRK-VI.2 or LecRK-VI.2 orthologs and possibly other LecRKs stand as promising candidates in the development of crops with durable, wide-range resistance.

## CONCLUSION AND PERSPECTIVES

Unlike R protein-mediated resistance that possesses narrow specificity, PTI is broad-spectrum and thus stands as a potential tool for engineering crops with enhanced immunity. Notably, interfamily transfer of genes encoding PRRs or key regulators of PTI enhances resistance of the recipient plant species against a broad range of virulent pathogens (**Figure 1**; Lacombe et al., 2010; Bouwmeester et al., 2014; Huang et al., 2014a; Tripathi et al., 2014). However, in some cases, such heterologous expression may lead to undesirable changes in growth and development (Bouwmeester et al., 2014). The emerging examples of interfamily transfer of PTI-related gene to confer broad-spectrum resistance is encouraging for the future development of resistant crops, but the durability and efficacy of this approach in the field is yet to be determined. In natural conditions, pathogens are constantly evolving to cope with host immunity (McDonald and Linde, 2002), and some pathogens acquire modified MAMPs to avoid recognition (Felix et al., 1999; Kunze et al., 2004; Lacombe et al., 2010). To achieve durable disease resistance, genetic-engineering should be used wisely, perhaps through stacking multiple PTI- and ETI-related genes, and proper field management should be deployed. The use of novel fundamental discoveries in PTI will definitively help the burgeoning of novel practical approaches to increase crop resistance to deleterious pathogens.

## Conflict of Interest Statement

The authors declare that the research was conducted in the absence of any commercial or financial relationships that could be construed as a potential conflict of interest.
